# MicroRNA expression signature and target prediction in familial and sporadic primary macronodular adrenal hyperplasia (PMAH)

**DOI:** 10.1186/s12902-021-00910-7

**Published:** 2022-01-05

**Authors:** Xiao-Gang Tan, Jie Zhu, Liang Cui

**Affiliations:** 1 Department of Thoracic Surgery, Xuan Wu Hospital of Capital Medical University, Beijing, 100053 China; 2 Department of Urology Surgery, Chinese PLA General Hospital, Beijing, 100082 China; 3 Department of Urology Surgery, Civil Aviation General Hospital, Beijing, 100123 China

**Keywords:** Primary macronodular adrenal hyperplasia (PMAH), microRNA expression, Familial, Sporadic, Pathway analysis, Pathogenesis

## Abstract

**Background:**

Primary macronodular adrenal hyperplasia (PMAH), previously termed ACTH-independent macronodular adrenal hyperplasia (AIMAH), is a rare cause of Cushing’s syndrome usually characterized by functioning adrenal macronodules and increased cortisol production.

**Methods:**

To screen and analyse the microRNA (miRNA) profile of PMAH in order to elucidate its possible pathogenesis, a miRNA microarray was used to test tissue samples from patients with familial PMAH, patients with sporadic PMAH and normal control samples of other nontumour adrenocortical tissues and identify characteristic microRNA expression signatures. Randomly selected miRNAs were validated by quantitative real-time reverse transcription polymerase chain reaction (qRT-PCR). Furthermore, the key signalling pathways and miRNAs involved in PMAH pathogenesis were determined by gene ontology and pathway analysis.

**Results:**

Characteristic microRNA expression signatures were identified for patients with familial PMAH (16 differentially expressed microRNAs) and patients with sporadic PMAH (8 differentially expressed microRNAs). The expression of the selected miRNAs was confirmed by qRT-PCR, suggesting the high reliability of the miRNA array analysis results. Pathway analysis showed that the most enriched pathway was the renal cell carcinoma pathway. Overexpression of miR-17, miR-20a and miR-130b may inhibit glucocorticoid-induced apoptosis in PMAH pathogenesis.

**Conclusion:**

We identified the miRNA signatures in patients with familial and sporadic PMAH. The differentially expressed miRNAs may be involved in the mechanisms of PMAH pathogenesis. Specific miRNAs, such as miR-17, miR-20a and miR-130b, may be new targets for further functional studies of PMAH.

## Background

ACTH-independent macronodular adrenal hyperplasia (AIMAH, OMIM #615954) is also called primary bilateral macronodular adrenal hyperplasia, and the term AIMAH has been recently suggested to be revised to primary macronodular adrenal hyperplasia (PMAH), which is a rare disorder characterized by bilateral macronodular hyperplasia of the adrenal glands with increased cortisol production. Patients typically present in the fifth and sixth decades of life with obvious clinical manifestations of moon face, sanguineous temperament, skin thinning, buffalo hump and supraclavicular fat pad thickening [1, 2]. PMAH accounts for fewer than 1% of cases of adrenocorticotropic hormone-independent Cushing’s syndrome, and the extent of cortisol excess ranges from subclinical to overt Cushing’s syndrome [3]. The production of cortisol in patients with PMAH is often aberrantly modulated by other hormones, such as vasopressin, gonadotropins, angiotensin, gastric inhibitory peptide and catecholamines, and endocrine disturbance is associated with abnormal adrenocortical sensitivity to various hormonal secretagogues. Furthermore, abnormal activity of a few hormone receptors has been considered to be a causative factor of PMAH [4, 5]. Although the disease phenotype occurs sporadically in the majority of affected patients with PMAH, several reports of familial clustering have been reported [6, 7]. Recently, with the development of gene sequencing technology, gene mutations related to familial and sporadic PMAH, such as ARMC5 mutations found by DNA sequencing [8] and whole-genome sequencing [9] and EDNRA mutations found by whole-exome sequencing [10], have been identified. PMAH presents a bimodal age distribution with a rare subset of patients presenting in the first years of life, particularly with McCune-Albright syndrome. Most patients with PMAH present in the fifth and sixth decades of life, a later age of onset than that of unilateral adenoma or Cushing’s syndrome [2, 11]. Although reports of large families with PMAH have suggested that the phenotype is consistent with a monogenetic, probable autosomal dominant inheritance pattern [12], the genetic or epigenetic basis of familial PMAH remains undetermined.

MicroRNA (miRNA)-associated research has generated great interest since the identification of the first microRNA owing to the extensive potential effects of miRNAs on the regulation of gene expression at both the transcriptional and posttranscriptional levels [13]. MicroRNAs are a class of single-stranded, noncoding small RNAs with a length of approximately 22 nucleotides [14, 15]. MiRNAs are first transcribed by RNA polymerase II in hairpin structures and are then processed by the RNase III Drosha to become long precursor miRNAs (pre-miRNAs). Then, the RNase III Dicer is involved in the processing from precursor to mature microRNAs, which can bind to the 3′ untranslated regions (3’UTRs) of their targeted messenger RNAs in a sequence-specific manner with complete or partial complementarity, leading to degradation or translational repression of the messenger RNA transcript, respectively [16]. The biological functions of only a small fraction of the identified miRNAs have been elucidated, and these miRNAs may be involved in essential regulatory processes such as proliferation, differentiation, apoptosis and development [17]. Although microRNA expression profiles differ among cancer types, it has been demonstrated that several microRNAs might play very specific roles in tumorigenesis by regulating a series of pathways [18–23].

**Fig. 1:**
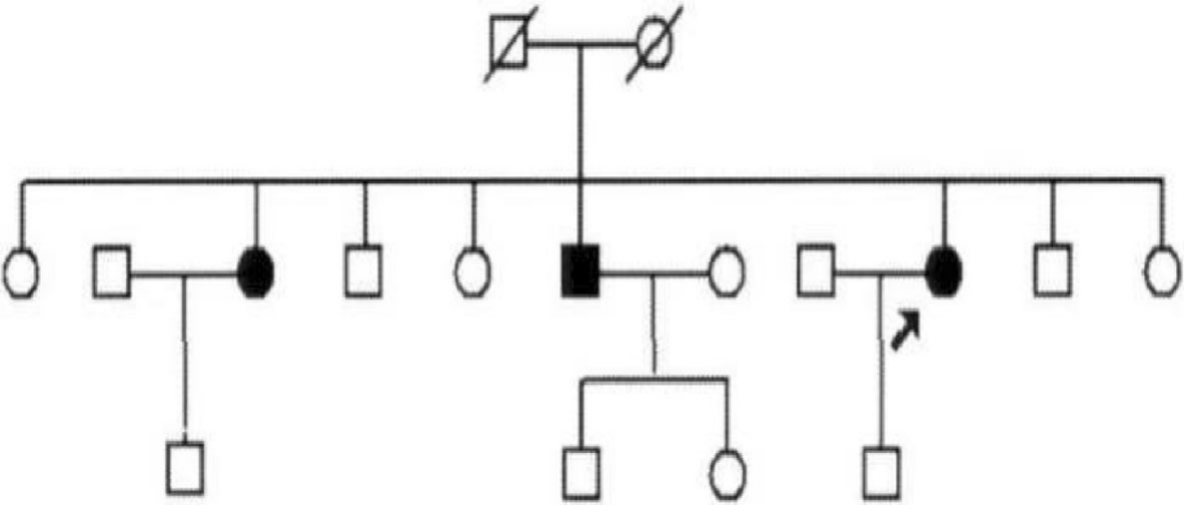
Pedigree of the familial patients with PMAH. Arrow: proband

PMAH is rare and generally presents as a sporadic disease. Familial clustering of PMAH is comparatively infrequent. In the past decade, PMAH-affected families have been diagnosed in the General Hospital of the People’s Liberation Army. Three out of seventeen members were diagnosed with typical or subclinical PMAH syndrome (Fig. 1). We collected samples from the affected patients from this familial pedigree and adrenal lipoma patients with complete clinical information by adrenalectomy. We previously performed whole-genome sequencing with samples from the three patients with familial PMAH [9]. The samples from the three patients were analysed to identify somatic mutations in ARMC5 exons. We believe that the microRNA expression signature may provide additional information to supplement the whole-genome analysis previously performed in this set of patients. The aims of the study were to identify differentially expressed miRNAs in the samples from patients with familial PMAH, patients with sporadic PMAH and normal control samples from adrenal lipoma patients via a high-throughput approach and to conduct bioinformatics analysis to determine the potential functions of the differentially expressed miRNAs and possible pathways mediating the pathogenesis of PMAH through Gene Ontology (GO) and Kyoto Encyclopedia of Genes and Genomes (KEGG) pathway analyses, respectively.

## Methods

### Clinical samples

**Fig. 2:**
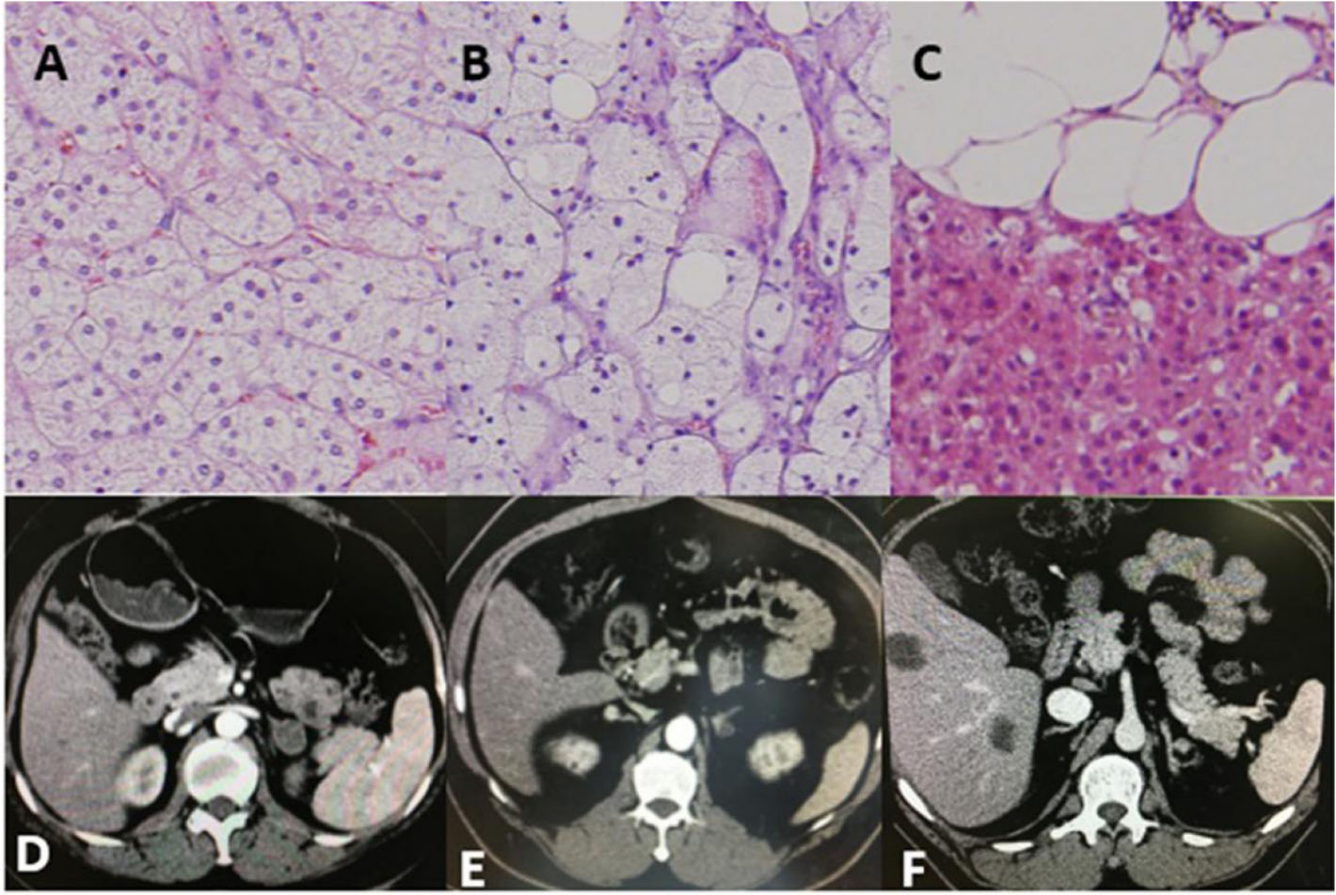
H&E staining (200X) and CT imaging. **A** Left adrenal nodule of familial PMAH H&E staining imaging. **B** Left adrenal nodule of sporadic PMAH H&E staining imaging. **C** Left adrenal lipoma of the normal control H&E staining imaging. **D** Left adrenal nodule of familial PMAH CT imaging. **E** Left adrenal nodule of sporadic PMAH CT imaging. **F** Left adrenal lipoma of the normal control CT imaging

All procedures were performed in accordance with the relevant guidelines and regulations. Written informed consent for genetic studies was obtained prior to initiating this study in agreement with protocols approved by the institutional review board at the General Hospital of the People’s Liberation Army. This research was reviewed and approved by the ethics committee of General Hospital of the People’s Liberation Army. Adrenal nodule samples from patients with familial and sporadic PMAH were collected after surgery. Normal control tissues were collected from the nontumour region of patients with large adrenal lipomas; this region was located ≥2 cm from the lipoma after surgery. Moreover, computed tomography (CT) imaging and haematoxylin and eosin (H&E) staining were employed to identify the characteristics of nodular hyperplasia. For CT analysis, the images were reviewed by two experienced observers who arrived at a consensus. The imaging characteristics were recorded. For H&E staining, all surgical specimens were fixed with 10% buffered formaldehyde and routinely processed for histologic diagnosis. The tumour histology was independently confirmed by 2 pathologists (Fig. 2). On the basis of the pathological diagnosis correction, 3 to 5 tissue pieces of approximately 3 mm^3^ according to the size of the lesion were cut and put into a 1 ml Eppendorf (EP) tube. The samples were snap frozen in liquid nitrogen immediately after resection and stored at − 80 °C until RNA extraction. We used one sample for whole-genome sequencing and another sample for microRNA profile screening.

### Total RNA extraction

Total RNA from the adrenal gland tissue removed from patients with familial or sporadic PMAH as well as normal control tissue was extracted according to the standard protocol.

### MicroRNA array analysis

An Affymetrix GeneChip miRNA Array (Affymetrix, Santa Clara, CA) was applied to investigate the differential microRNA expression profile in familial and sporadic PMAH samples compared with normal control samples. This microRNA array contained 46,228 probes, which composes 7815 probe sets, including controls, and covers 71 organisms, including humans, mice, rats, canines and rhesus macaques. The array content was derived from the Sanger miRBase miRNA database v.11 (April 15, 2008). In brief, 1.5 μg of total RNA extracted from different groups of samples was labelled using a 3DNA Array Detection FlashTag™ RNA Labeling Kit according to the recommendations of the manufacturer. First, the addition of a poly(A) tail was carried out at 37 °C for 15 min in a 15 μl reaction mixture, which contained 1× Reaction Buffer, 1.5 ml of MgCl_2_, 1 μl of ATP Mix diluted 1:500 and 1 μl of PAP enzyme. Second, flash tag ligation was performed at room temperature for 30 min by adding 4 μl of 5× Flash Tag Ligation Mix Biotin and 2 μl of T4 DNA ligase into the 15 μl reaction mixture. To stop the reaction, 2.5 μl of Stop Solution was added. All samples were hybridized, washed and scanned with an Affymetrix Scanner.

### MiRNA qRT-PCR

Reverse transcription (RT) was performed on miRNAs using a TaqMan MicroRNA Reverse Transcription Kit (Applied Biosystems) with incubation at 16 °C for 30 min, 42 °C for 30 min, and 85 °C for 5 min for denaturation of the enzyme. RT was performed at 37 °C for 1 h followed by 5 min at 95 °C. TaqMan microRNA assays (Applied Biosystems) were performed using a 7500 Fast Real-Time PCR System in the “9600 emulation” run mode. Ct values were converted into copy numbers (copy no. = 2^(−Ct)) and normalized to RNU48.

### Bioinformatics analysis

The GO Project provides a controlled vocabulary to describe genes and gene products in any organism (http://www.geneontology.org). The ontology covers three categories: biological process, cellular component and molecular function. Fisher’s exact test was used to determine if there was more overlap between the differentially expressed gene list and the GO annotation list than would be expected by chance. The *P*-value denotes the significance of the enrichment of a GO term with the differentially expressed genes. The lower the *P*-value, the more significant is the GO term (a P-value < 0.05 is the recommended cut-off criterion).

Pathway analysis is a functional analysis that maps genes to KEGG pathways. The *P*-value (EASE score, Fisher P-value or hypergeometric P-value) denotes the significance of a pathway correlated to the conditions. The lower the P-value, the more significant is the pathway (the recommended cut-off P-value is 0.05).

### Statistical analysis

The results are expressed as the mean ± SD values. Data for haemodynamic parameters were compared using paired t-tests. Values of *P* < 0.05 were considered statistically significant (P < 0.05 for differentially expressed miRNAs).

For comparing the differentially expressed miRNA profiles between two groups, fold change values and *P*-values were calculated and used to identify significantly differentially expressed miRNAs (based on the all-isoform value). Differentially expressed miRNAs between two samples were defined by filtering with a fold change criterion (based on the all-isoform value), and hierarchical clustering was then performed. MiRNA targets were predicted by GO analysis, and KEGG pathway analysis was performed based on the differentially expressed miRNAs.

## Results

### Expression profiles of microRNAs in patients with familial or sporadic PMAH compared with normal controls

**Fig. 3:**
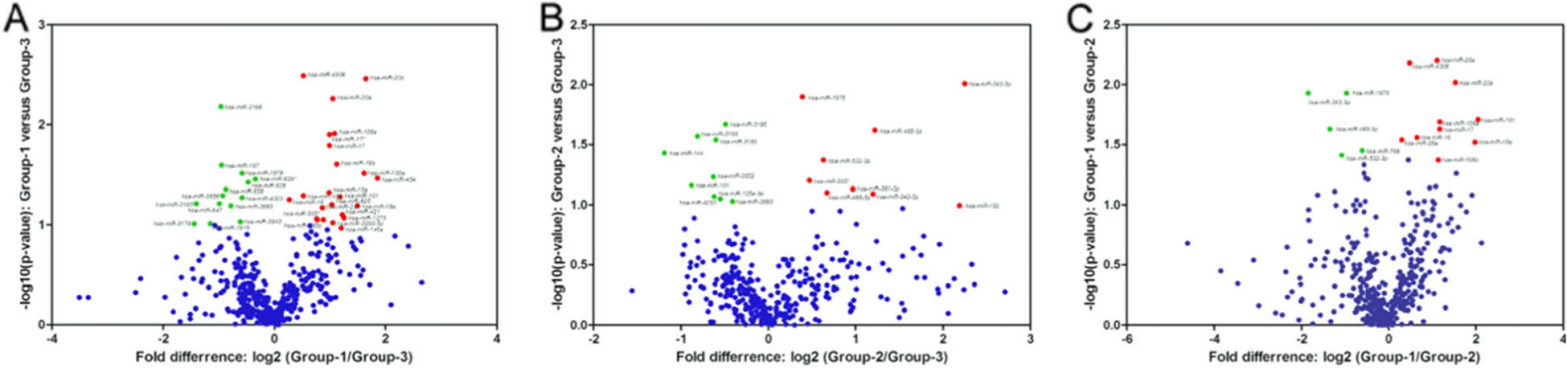
Volcano plot demonstrating differences in expression levels of microRNAs in 2 groups of PMAH patients and normal controls based on the microarray study. The log-fold changes were plotted based on the log odds of differential expression. The microRNAs with significant differences in expression levels (p<0.05) between familial patients with PMAH and normal controls after Benjamini-Hochberg correction are indicated in (**A**). The microRNAs with significant differences in expression levels (p<0.05) between sporadic patients with PMAH and normal controls after correction are indicated in (**B)**. The microRNAs with significant differences in expression levels (p<0.05) between familial and sporadic patients with PMAH after correction are indicated in (**C**)

A microRNA microarray analysis was performed on tissue samples from 3 patients with familial PMAH (Group 1), tissue samples from 2 patients with sporadic PMAH (Group 2) and 3 normal control tissues (Group 3) to compare the differences in microRNA expression levels. The differences in microRNA expression were analysed between the patients with familial PMAH and the normal controls. A total of 16 microRNAs met the false discovery rate (FDR) cut-off criterion of 0.05. Seven miRNAs were upregulated (hsa-miR-4306, hsa-miR-130a, hsa-miR-20b, hsa-miR-20a, hsa-miR-15a, hsa-miR-106a, and hsa-miR-17), and 3 miRNAs were downregulated (hsa-miR-197, hsa-miR-3656, and hsa-miR-3196) (Table 1). However, the following microRNAs exhibited statistically significant differential expression, albeit with very low signal intensities: hsa-miR-17*, hsa-miR-18b, hsa-miR-1976, hsa-miR-454, hsa-miR-629*, and hsa-miR-629. After filtering out the low-intensity miRNAs, the raw signal intensities were normalized to the median value. The differentially expressed miRNAs passed volcano plot filtering (Fig. 3A).

**Table 1:** MiRNAs with significantly different expression (*P* < 0.05) between familial patients with PMAH (Group1) and normal control tissues (Group3)

miRNAs	Fold change	*P* value	Case/control
hsa-miR-4306	1.43	0.003223	Up
hsa-miR-130a	3.05	0.03120	Up
hsa-miR-20b	3.11	0.003463	Up
hsa-miR-20a	2.07	0.005487	Up
hsa-miR-15a	1.97	0.04733	Up
hsa-miR-106a	1.98	0.01258	Up
hsa-miR-17	1.98	0.01607	Up
hsa-miR-197	0.52	0.02572	Down
hsa-miR-3656	0.53	0.04458	Down
hsa-miR-3196	0.51	0.006570	Down
hsa-miR-17*		0.01237	Up
hsa-miR-18b		0.02489	Up
hsa-miR-629		0.03823	Up
hsa-miR-1976		0.03086	Down
hsa-miR-629*		0.03518	Down
hsa-miR-454		0.03496	Down

A similar analysis was performed to assess differences in microRNA expression levels between 2 patients with sporadic PMAH and 3 normal controls. Through filtering according to a FDR cut-off criterion of 0.05 and microRNA expression signal intensities, 8 microRNAs were found to be differentially expressed between the above 2 groups; 1 miRNA was upregulated (hsa-miR-3196) and 2 miRNAs were downregulated (hsa-miR-342-3p and hsa-miR-532-3p) (Table 2). After filtering out the low-intensity miRNAs, the raw signal intensities were normalized to the median value. The differentially expressed miRNAs passed volcano plot filtering (Fig. 3B). The other microRNAs showed no differences in expression between patients with sporadic PMAH and normal controls.

**Table 2:** MiRNAs with significantly different expression (P < 0.05) between sporadic cases (Group2) and normal control tissues (Group3)

miRNAs	Fold change	P value	Case/control
hsa-miR-3196	1.41	0.02156	Up
hsa-miR-342-3p	0.21	0.009685	Down
hsa-miR-532-3p	0.65	0.04271	Down
hsa-miR-3195		0.0272	Up
hsa-miR-3180		0.0288	Up
hsa-miR-445		0.0370	Up
hsa-miR-1976		0.0125	Down
hsa-miR-485-3p		0.0241	Down

To identify the microRNAs that were differentially expressed between patients with familial and sporadic PMAH, we compared the microRNA expression profiles of these two independent groups of samples. The same criteria for the FDR cut-off value and expression intensity were applied to filter the candidate microRNAs. Two miRNAs were upregulated (hsa-miR-342-3p and hsa-miR-532-3p), and 9 miRNAs were downregulated (hsa-miR-4306, hsa-miR-20b, hsa-miR-20a, hsa-miR-106a, hsa-miR-17, hsa-miR-101, hsa-miR-16, hsa-miR-26a, and hsa-miR-106b) (Table 3). After filtering out the low-intensity miRNAs, the raw signal intensities were normalized to the median value. The differentially expressed miRNAs passed volcano plot filtering (Fig. 3C).

**Table 3:** MiRNAs with significantly different expression (P < 0.05) between familial patients with PMAH (Group1) and sporadic cases (Group2)

miRNAs	Fold change	P value	Case/control
hsa-miR-342-3p	3.6	0.01188	Up
hsa-miR-532-3p	2.1	0.03911	Up
hsa-miR-4306	0.71	0.006598	Down
hsa-miR-20b	0.35	0.009508	Down
hsa-miR-20a	0.46	0.006258	Down
hsa-miR-106a	0.44	0.04284	Down
hsa-miR-17	0.44	0.02335	Down
hsa-miR-101	0.24	0.01947	Down
hsa-miR-16	0.64	0.02747	Down
hsa-miR-26a	0.81	0.02877	Down
hsa-miR-106b	0.46	0.02046	Down

### Validation of microRNA array analysis results by qRT-PCR

To validate the microRNA microarray analysis results, qRT-PCR was performed on total RNA extracted from surgical tissues obtained from patients with familial and sporadic PMAH and surgical tissues obtained from normal controls. The expression level of hsa-miR-20b in patients with familial PMAH was 1.56-fold higher than that in normal controls, with a *P*-value of 0.01165, consistent with the microRNA array analysis results (3.11-fold higher, P-value = 0.003463). The expression level of hsa-miR-342-3p (downregulated 0.68-fold with P-value = 0.005814) was consistent with the result from the microRNA array analysis (hsa-miR-342-3p was downregulated 0.21-fold in patients with sporadic PMAH compared with normal controls). Hsa-miR-342-3p was upregulated 1.58-fold but hsa-miR-101 was downregulated 0.40-fold in patients with familial PMAH compared to patients with sporadic PMAH, consistent with the microRNA array analysis results, in which hsa-miR-342-3p was upregulated 3.6-fold and hsa-miR-101 was downregulated 0.24-fold in patients with familial PMAH compared with those with sporadic PMAH.

### Hierarchical clustering of microRNAs with differential expression

**Fig. 4:**
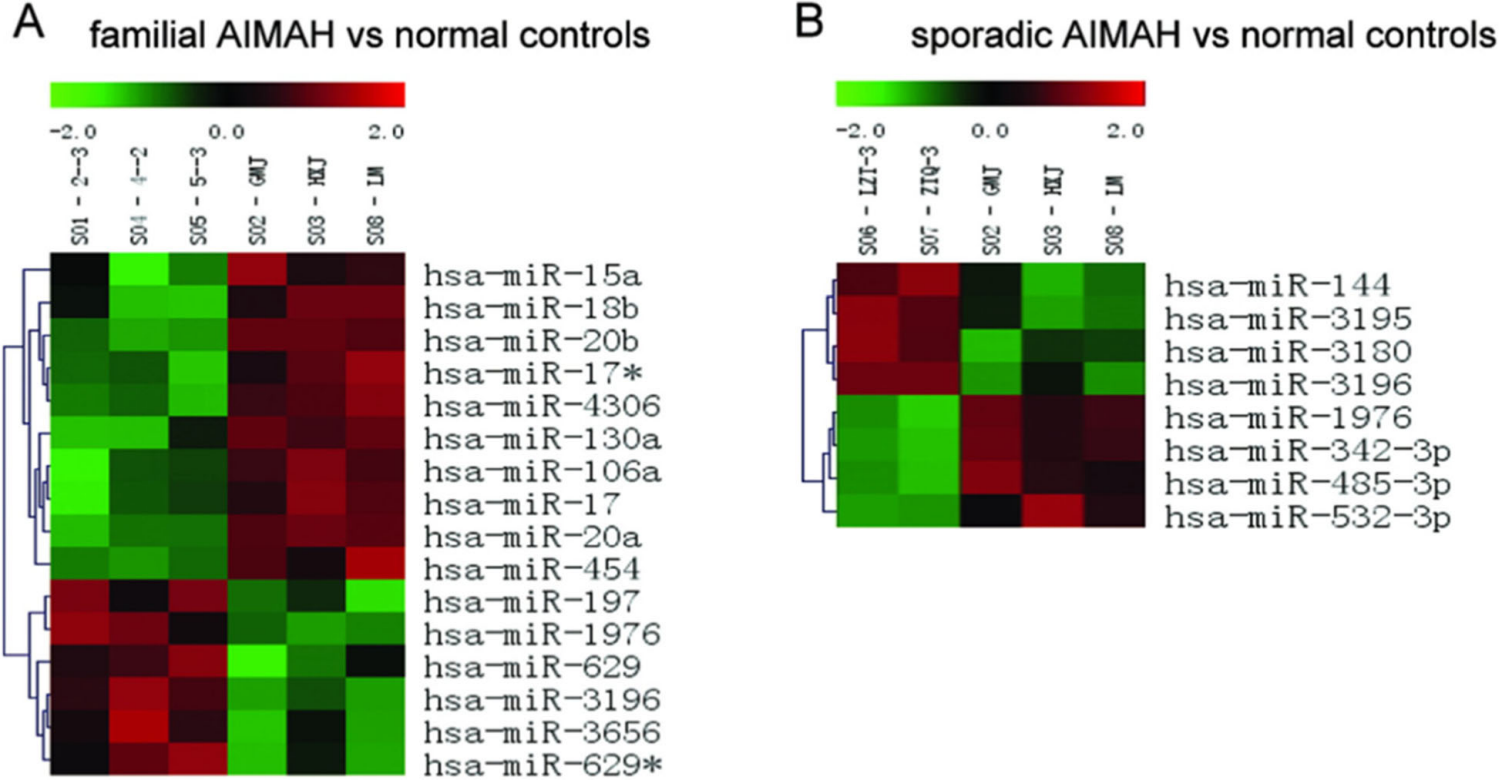
microRNAs expression signature consisting of diffrentially expressed microRNAs between familial or sporadic PMAH and normal controls. A. Differentially expressed microRNAs (p<0.05) were clustered and the results showed that the expression profiles of these 16 microRNAs could be used to separate between the familial PMAH and normal controls. B. Differentially expressed microRNAs (p<0.05) were clustered and the results showed that the expression profiles of these 8 microRNAs could be used to separate between the sporadic PMAH and normal controls

To identify the unique microRNA expression signatures among different groups of patients, hierarchical clustering analysis was performed on all microRNAs based on the expression signal intensities obtained by microarray analysis. This hierarchical clustering analysis clustered microRNAs together according to their expression levels and clustered samples from different groups based on the similarity of the expression profiles for the investigated microRNAs [24]. By supervised hierarchical clustering with pairwise comparisons among these 3 groups of samples, we could always identify a unique group of microRNAs that were able to differentiate one group from the other. The 16 differentially expressed microRNAs (10 downregulated and 6 upregulated microRNAs) in the signature identified by microarray analysis clearly discriminated between patients with familial PMAH and normal controls (Fig. 4A). Similarly, the microRNA signature containing 8 differentially expressed microRNAs were able to discriminate between patients with sporadic PMAH and normal controls with a statistically significant *P*-value of 0.05 (Fig. 4B). The 11 identified differentially expressed microRNAs were able to discriminate between patients with familial PMAH and patients with sporadic PMAH (Table 3).

### Comprehensive target-network prediction and pathway analysis of differentially expressed microRNAs

**Fig. 5:**
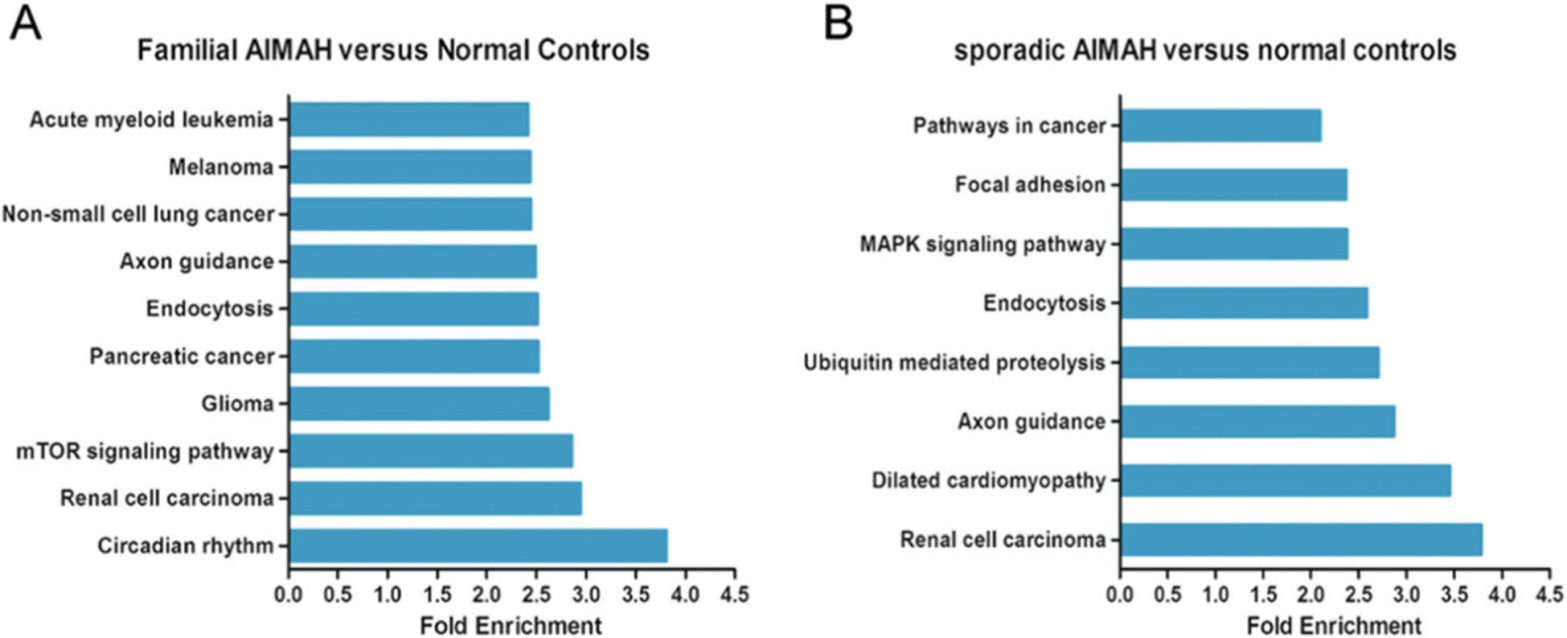
KEGG pathway analysis on diffrentially expressed microRNAs between familial or sporadic PMAH and normal controls. A. KEGG Pathway analysis was performed on 16 altered microRNAs to reveal that Circadian Rhythm pathway was the most affected pathway. B. KEGG Pathway analysis was performed on 8 altered microRNAs to reveal that renal cell carcinoma pathway was ranked the most affected pathway

Functional analyses of the differentially expressed microRNAs between the PMAH patients and normal controls revealed that some enriched pathways, such as signal transduction, signalling molecules and metabolic interactions, were potentially associated with the pathogenesis of PMAH. For comparative analysis between patients with familial PMAH and normal controls, all 16 differentially expressed microRNAs were imported into KEGG pathway analysis software, and the circadian rhythm pathway was the pathway most affected by these microRNAs, containing 6 genes predicted to be potential targets (NPAS2, CRY2, BHLHE40, BHLHE41, CRY1 and CLOCK). However, the renal cell carcinoma pathway, mTOR signalling pathway, glioma pathway, pancreatic cancer pathway and endocytosis pathway were the top signalling pathways affected by these differentially expressed microRNAs (Fig. 5A). When KEGG pathway analysis was similarly performed on the 8 differentially expressed microRNAs between the patients with sporadic PMAH and normal controls, the renal cell carcinoma, dilated cardiomyopathy, axon guidance, ubiquitin-mediated proteolysis, endocytosis and MAPK signalling pathways were ranked as the most affected pathways on the basis of the predicted targets, while the renal cell carcinoma pathway contained 5 genes (CDC42, CUL2, EP300, GRB2 and SLC2A1) that were predicted to be potential downstream targets of the differentially expressed microRNAs (Fig. 5B).

**Fig. 6:**
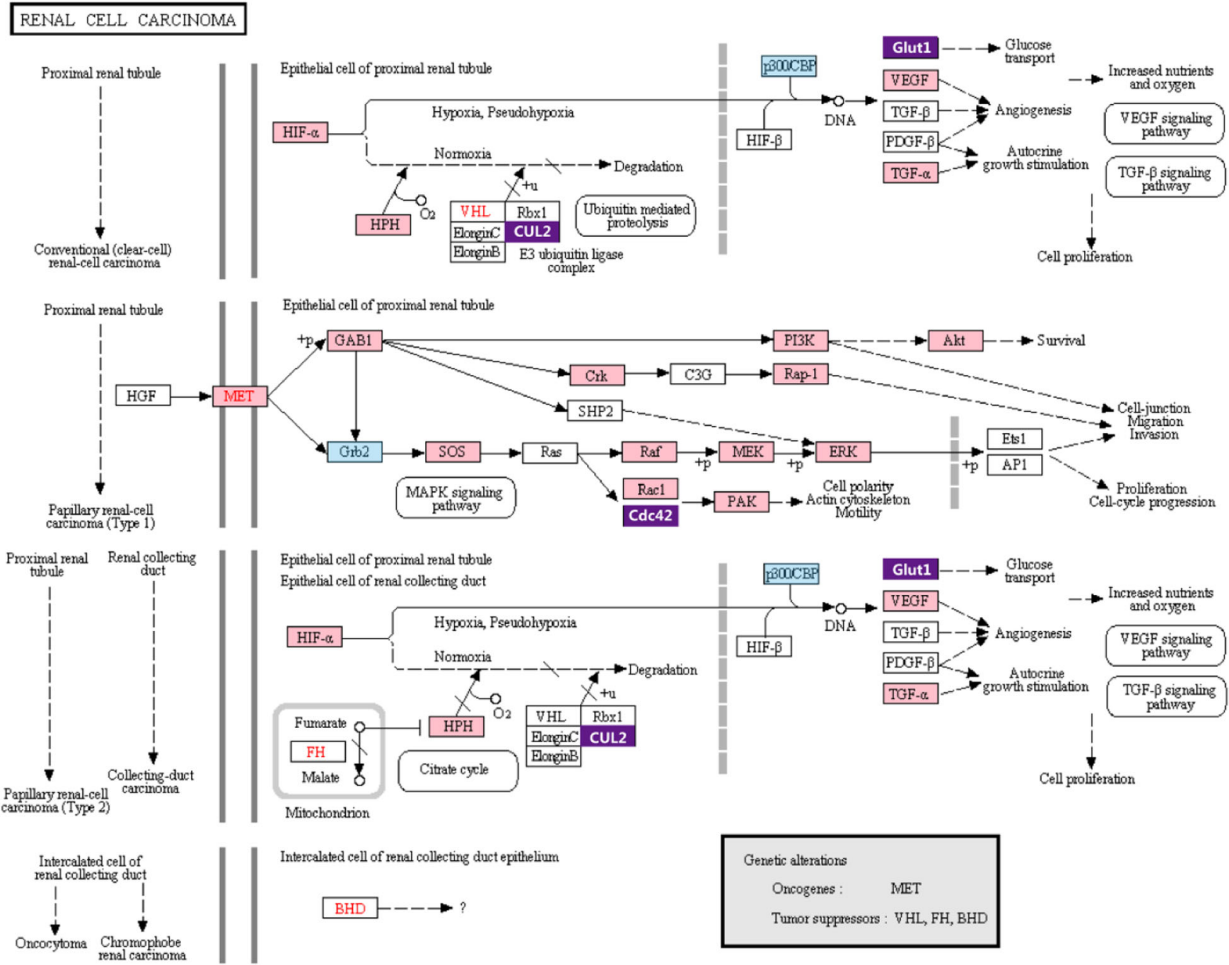
Renal cell carcinoma pathway was predicted to be target of microRNAs differentially expressed between PMAH patients and normal controls. The pink rectangles were indicated to be targets of microRNAs diffrentially expressed between familial patients with PMAH and normal controls, the blue rectangles were targets of microRNAs diffrentially expressed between sporadic patients with PMAH and normal controls, the purple rectangles were common targets of the 2 groups of differentially expressed microRNAs

Interestingly, the renal cell carcinoma pathway was one of the top 2 most affected pathways among all associated pathways containing potential targets of the microRNAs that were differentially expressed in patients with either familial or sporadic PMAH compared to normal controls. In the comparison of differentially expressed microRNAs between patients with familial PMAH and normal controls, the renal cell carcinoma pathway contained 25 genes (EGLN3, EGLN2, EGLN1, PAK6, PAK7, CUL2, CDC42, RAC1, SOS2, GAB1, SLC2A1, TGFA, PAK1, PIK3R1, AKT3, PIK3R2, MAP 2 K1, MET, RAF1, MAPK1, HIF1A, CRKL, VEGFA, RAP1A and CRK) that were targeted by these microRNAs, while 5 genes (CDC42, CUL2, EP300, GRB2 and SLC2A1) were targeted by microRNAs that were differentially expressed between patients with sporadic PMAH and normal controls (Fig. 6).

## Discussion

Recent studies have indicated that microRNA expression signatures might be useful for the characterization and prediction of certain diseases, especially benign or malignant tumours [25, 26], but the investigation of microRNA expression signatures in patients with familial PMAH and sporadic PMAH, which have very low incidence rates, remains unclear. In this study, microRNA microarray analysis was applied to identify the differentially expressed microRNAs between patients with familial or sporadic PMAH and normal controls. Our results revealed that a series of differentially expressed microRNAs found by comparisons of different groups could contribute to the characteristics of PMAH.

Before the experiments, the individual performing quality control on the miRNA microarray analysis results randomly selected hsa-miR-20b, hsa-miR-342-3p and hsa-miR-101 for qRT-PCR to validate the microRNA microarray analysis results. We evaluated the expression level of hsa-miR-20b, which, based on our microarray data, was differentially expressed in tissues from patients with familial PMAH compared with normal controls. The expression level of hsa-miR-20b in patients with familial PMAH was 1.56-fold higher than that in normal controls, consistent with the microRNA array analysis results. The expression level of hsa-miR-342-3p was determined by qRT-PCR in samples from patients with sporadic PMAH and normal controls to validate the comparison of the microRNA expression profile between these 2 groups, and the results were consistent with those of the microRNA array analysis. Similarly, hsa-miR-342-3p and hsa-miR-101 were selected to validate the differential microRNA expression levels between patients with familial PMAH and patients with sporadic PMAH. The qRT-PCR results showed that hsa-miR-342-3p was upregulated 1.58-fold but hsa-miR-101 was downregulated 0.40-fold in patients with familial PMAH compared to patients with sporadic PMAH. These qRT-PCR results were consistent with those of the microRNA array analysis, in which hsa-miR-342-3p was upregulated 3.6-fold and hsa-miR-101 was downregulated 0.24-fold in patients with familial PMAH compared with those with sporadic PMAH. Hsa-miR-101, hsa-miR-20b and hsa-miR-342-3p were validated by qRT-PCR, and the results were directly correlated with our microRNA microarray data, suggesting the high reliability of the miRNA array analysis results.

KEGG pathway analysis of the selected microRNAs and further validation by qRT-PCR revealed that these microRNAs had putative downstream targets involved in many important pathways, including the circadian rhythm pathway, renal cell carcinoma pathway, MAPK signalling pathway and other signalling pathways.

In our study, the renal cell carcinoma pathway was ranked as the most affected signalling pathway in both patients with familial PMAH and patients with sporadic PMAH compared to normal controls, although PMAH is considered to be a cytologically benign disease without a propensity for invasion and metastasis. In addition, CUL2, CDC42, GLUT1 and other proteins, which have been demonstrated to be critical in malignant renal cell carcinoma, were predicted to be downstream target proteins of microRNAs differentially expressed in PMAH patients compared with normal controls. Our findings in this study were consistent with previous studies from other groups; for instance, the microRNA expression profile for massive macronodular adrenocortical disease shared differentially expressed microRNAs with that for renal cell carcinoma, and these microRNAs were previously implicated in tumorigenesis or metastasis [27]. In another study, several aberrantly expressed genes that were demonstrated to be involved in oncogenic pathways were also identified in one PMAH patient by gene expression profiling [28]. Although PMAH is considered benign because metastasis and invasion have never been reported in long-term postoperative surveillance of patients [29], a massive increase in the adrenocortical cell mass is a typical characteristic of PMAH. The abovementioned results indicate an abnormality in the control of proliferation in adrenocortical cells, which is similar to the characteristics of malignant tumors to some extent. The potential mechanisms underlying abnormalities cell apoptosis might at least partially include the disruption of normal cell cycle progression through alterations in microRNA expression.

MiR-17 and miR-20a, which were significantly upregulated in patients with familial PMAH compared with normal controls, are two of the six miRNAs in the miR-17-92 cluster (which contains miR-17, −18a, −19a, −19b-1, −20a, and -92a-1) generated from a single precursor RNA that is transcribed from chromosome 13 [30]. Both miR-17 and miR-20a have been reported to contribute to glucocorticoid-induced apoptosis of chondrocytes in lymphoma [31–33]. MiR-17 has been reported to be involved via the miR-17 pathway in glucocorticoid-induced cell death in paediatric acute lymphoblastic leukaemia [34–36]. The NR3C1-targeting miR-130b was found to exhibit higher expression in a glucocorticoid-insensitive multiple myeloma cell line, and its introduction into a glucocorticoid-sensitive line impaired the cellular response to glucocorticoid treatment, including the induction of apoptosis [37]. Therefore, we might predict that overexpression of miR-17, miR-20a and miR-130b can inhibit glucocorticoid-induced apoptosis in PMAH pathogenesis.

Hsa-miR-342-3p, hsa-miR-20b and hsa-miR-101, targeting CDC42, ERK and MEK, respectively, in the MAPK signalling pathway or abnormal expression of PI3K/AKT mediated by differentially expressed microRNAs might interfere with normal apoptosis progression, in turn promoting the survival of adrenocortical cells. In addition, the TGF-β signalling pathway and VEGF signalling pathway are involved in the pathogenesis of PMAH.

In the comparison between familial PMAH patients and normal controls, the circadian rhythm pathway was the most affected signalling pathway based on the downstream targets of the differentially expressed microRNAs. Circadian rhythms are universal 24-h oscillation patterns in the metabolic, physiological, endocrinal and behavioural functions of almost all species, and core circadian genes such as PER, CRY and CLOCK seem to be important for tissue homeostasis and tumorigenesis [38–40]. In endocrine diseases, key components of the circadian rhythm pathway can interact with steroid hormone receptors to influence the pathogenesis of some endocrine tumours. Core circadian rhythm proteins such as PER2 and CLOCK can be coexpressed with steroid hormone receptors, and CLOCK-associated genes can regulate glucocorticoid activity in almost all tissues by enhancing the transcriptional activity of glucocorticoid receptors (GRs) [41, 42]. One potential underlying mechanism involves acetylation of several lysine residues on GRs and concomitant attenuation of GR binding to glucocorticoid response elements [43]. Because PMAH is characterized by bilateral massive enlargement with dysfunction of glucocorticoid hormones as well as their response receptors, circadian rhythm pathways might play an important role during the development of this disease through their interactions with glucocorticoid receptors.

## Conclusion

Analysis of microRNA expression signatures revealed that the renal cell carcinoma pathway might play an important role in the pathogenesis of PMAH, although PMAH is a benign hereditary endocrine abnormality without malignant characteristics. Specific microRNAs, such as miR-17, miR-20a and miR-130b, play a role in the pathogenesis and progression of PMAH, and our findings contribute not only to an improved understanding of this benign disease but also to the development of new therapeutic and preventative strategies for PMAH.

## Data Availability

The data that support the findings of this study are available from the corresponding author upon reasonable request.

## Abbreviations

PMAH: Primary macronodular adrenal hyperplasia
miRNA: Microrna
qRT-PCR: Quantitative real-time reverse transcription polymerase chain reaction
GO: Gene Ontology
KEGG: Kyoto Encyclopedia of Genes and Genomes
CT: The computed tomograph
H&E staining: Hematoxylin-eosin staining
